# Keeping it simple: studying grammatical encoding with lexically reduced item sets

**DOI:** 10.3389/fpsyg.2014.00783

**Published:** 2014-07-18

**Authors:** Alma Veenstra, Daniel J. Acheson, Antje S. Meyer

**Affiliations:** ^1^Psychology of Language Department, Max Planck Institute for PsycholinguisticsNijmegen, Netherlands; ^2^Donders Institute for Brain, Cognition, and BehaviourNijmegen, Netherlands; ^3^Radboud UniversityNijmegen, Netherlands

**Keywords:** language production, number agreement, subject–verb agreement, grammatical number, grammatical encoding, number attraction, attraction asymmetry

## Abstract

Compared to the large body of work on lexical access, little research has been done on grammatical encoding in language production. An exception is the generation of subject-verb agreement. Here, two key findings have been reported: (1) speakers make more agreement errors when the head and local noun of a phrase mismatch in number than when they match [e.g., *the key to the cabinet(s)*]; and (2) this attraction effect is asymmetric, with stronger attraction for singular than for plural head nouns. Although these findings are robust, the cognitive processes leading to agreement errors and their significance for the generation of correct agreement are not fully understood. We propose that future studies of agreement, and grammatical encoding in general, may benefit from using paradigms that tightly control the variability of the lexical content of the material. We report two experiments illustrating this approach. In both of them, the experimental items featured combinations of four nouns, four color adjectives, and two prepositions. In Experiment 1, native speakers of Dutch described pictures in sentences such as *the circle next to the stars is blue*. In Experiment 2, they carried out a forced-choice task, where they read subject noun phrases (e.g., *the circle next to the stars*) and selected the correct verb-phrase (*is blue* or *are blue*) with a button press. Both experiments showed an attraction effect, with more errors after subject phrases with mismatching, compared to matching head and local nouns. This effect was stronger for singular than plural heads, replicating the attraction asymmetry. In contrast, the response times recorded in Experiment 2 showed similar attraction effects for singular and plural head nouns. These results demonstrate that critical agreement phenomena can be elicited reliably in lexically reduced contexts. We discuss the theoretical implications of the findings and the potential and limitations of studies using lexically simple materials.

## INTRODUCTION

In order to produce phrases and sentences, speakers need to select words from their mental lexicon and combine them according to the grammatical rules of their language. Compared to the substantial body of work on lexical access, grammatical encoding processes have received little attention. In part, the relative neglect in investigating grammatical encoding may be due to methodological reasons. It is much easier to elicit specific words (e.g., nouns by using a picture naming task) than specific sentence structures. The main goal of the present paper is to illustrate that basic grammatical encoding processes can be investigated using paradigms and materials that are hardly more complex than those typically used in studies of single word production. Moreover, we argue that using very simple and uniform materials may often be beneficial in studies of grammatical encoding because it minimizes random variance in the participants’ responses due to irrelevant variability in lexical content. The experiments illustrating this research strategy concern subject–verb agreement. Before describing them, we review how grammatical agreement has been studied to date and discuss two of the main findings of these earlier studies.

In many languages, including English and Dutch, the main verb agrees in number with the subject of the sentence. In principle, the rule is simple: singular subjects require singular verbs and plural subjects require plural verbs. Subject–verb agreement is computed for almost every sentence we utter, and as it is implemented so frequently, the process is usually fast and errorless. However, sometimes speakers make errors where the number of the verb does not agree with the number of the subject (Bock and Miller, 1991; Bock and Eberhard, 1993; Vigliocco et al., 1995; Bock et al., 1999; Haskell and MacDonald, 2005). These errors provide a window into the process of agreement and enable researchers to study how conceptual information is mapped onto linguistic representations. The main tool in research on subject–verb agreement has been to elicit agreement errors, typically by presenting participants with complex subject–noun phrases (e.g., *The key to the cabinets*), and asking them to provide a verb phrase to complete a sentence (e.g., *are missing*; Bock and Miller, 1991).

In the first study to induce agreement errors experimentally, Bock and Miller (1991) presented participants with subject phrases such as *the key to the cabinets*. Participants listened to the subject phrase, repeated it, and added a verb phrase to complete the sentence (e.g., *the key to the cabinets is missing*). A much replicated central finding of this study has been dubbed attraction: it is the observation that in sentences starting with complex noun phrases, agreement errors are more likely when a local noun (i.e., *cabinets* in the above example) mismatches in number with the head noun (i.e., *key*), relative to when the two nouns match in number (as in *the key to the cabinet*). This attraction effect indicates that the head noun and the local noun in some way compete for control of the number specification of the verb.

A second key finding of Bock and Miller’s (1991) study was that the attraction effect was stronger for phrases with singular heads [e.g., *the key to the cabinet(s)*] than for phrases with plural heads [e.g., *the keys to the cabinet(s)*]. This attraction asymmetry has been replicated in numerous studies (Bock and Miller, 1991; Bock and Eberhard, 1993; Vigliocco et al., 1995; Bock et al., 1999; Haskell and MacDonald, 2005; but see Franck et al., 2002, 2006), and has been related to the morphological marking of number (e.g., Bock and Eberhard, 1993; Bock, 2004; Berent et al., 2005; Eberhard et al., 2005). Plural nouns possess an overt plural marker (*-s* in English, *-s* or *-en* in Dutch), which singular nouns do not possess (but see Corbett, 2000, for languages that mark both singular and plural). To explain the asymmetry in the patterns of agreement errors, it has been proposed that plural local nouns, due to their plural marking, can bias the computation of the number of the subject noun phrase and the selection of the verb form toward plurality, whereas singular local nouns, which are unmarked for number, cannot bias these processes in the opposite direction. Evidence consistent with this view comes from Eberhard (1997), who found that attraction from a plural local noun was diminished when the singular head noun was explicitly marked for number (e.g., *one key to the cabinets*), and that attraction from a singular local noun increased when the singular local noun was explicitly marked for number (e.g., *the keys to one cabinet*). This is in line with the view that singulars are unmarked by default and need explicit number marking to create attraction.

In Bock and Miller’s (1991) study, participants were free to complete the sentences in any way they wished. This led to high rates of responses that could not be scored (close to 40% in Experiments 1 and 2, almost 75% in Experiment 3) because the subject phrase was repeated incorrectly or the verb was uninflected (e.g., a past tense form). To limit the number of invalid responses, later studies have restricted the ways in which participants could complete the sentences. For instance, participants were presented with adjectives or past participles (e.g., *old* or *broken*) that had to be used in the completion together with an inflected form of *to be*, which increased the number of analyzable responses (Vigliocco et al., 1996; Barker et al., 2001; Haskell and MacDonald, 2003; Hartsuiker and Barkhuysen, 2006; Brehm and Bock, 2013; Veenstra et al., 2014a). Other studies encouraged the use of forms of *to be* by presenting infinitive verbs that had to be used in passive constructions (Hartsuiker et al., 2001), or verb stems to be used in perfect tense constructions (Thornton and MacDonald, 2003), or by simply instructing participants to use *to be* (Franck et al., 2002).

Further refining agreement paradigms, some studies have included response times as an additional dependent measure. Haskell and MacDonald (2003) presented participants with subject phrases and asked them to form questions using these phrases. As questions often start with inflected verbs, response onset latencies indicate the time needed to produce agreement. Importantly, this study demonstrated that the latencies for correct responses were longer in conditions that usually yield more agreement errors. Similarly, Brehm and Bock (2013) instructed participants to read the preambles silently and produce only the completions aloud as fast as possible. They found that the delay between the end of the visual presentation of the subject phrase and the onset of the response was longer for mismatching than for matching head and local nouns.

Finally, Staub (2009, 2010) developed a paradigm where participants were not required to produce the verb phrases but simply had to select one of two verb forms in a forced-choice task. Here, participants read subject phrases word by word on a computer screen, followed by a screen that showed the singular verb *is* on the left and plural verb *are* on the right. Participants had to press a left or right key as fast as possible for the option they thought would be the best continuation of the subject phrase. Again, longer response times were found for preambles with mismatching than with matching nouns. Veenstra et al. (2014a) used this paradigm and the paradigm used by Brehm and Bock (2013) with the same set of items and found comparable patterns of results for both, suggesting that both capture comparable aspects of the agreement process.

In the sentence completion experiments described so far, the materials were carefully matched across conditions, typically by showing different versions of the same noun phrase [e.g., *the bridge to the island(s)*] to different groups of participants. Within experimental conditions, items varied in lexical content, and repetitions of head or local nouns were avoided. This variation gives the materials a certain ecological validity, and has the benefit of potentially increasing the interest of the task for the participant, disguising the research questions, and preventing participants from developing *ad hoc* strategies. Moreover, if the goal of a study is to investigate how grammatical and semantic variables jointly affect agreement, both the syntactic structure and the lexical content of the items need to be varied.

For many purposes, however, it is not necessary, or even desirable, to disguise the purpose of a test, or to introduce variability across items. For instance, tests of vocabulary, arithmetic skills, and working memory are typically presented to participants without any disguise. These tests are designed in such a way that the impact of irrelevant skills (e.g., knowledge of the grammar when vocabulary is at stake) is minimized and that variability across items and across participants can be attributed primarily to relevant, experimentally controlled variables. For instance, researchers studying lexical access in production typically reduce the difficulty and variability of grammatical encoding processes to a minimum by presenting single words (Schriefers et al., 1990; Levelt et al., 1999; Damian et al., 2001; Ferreira and Pashler, 2002). Similarly, researchers studying morphology have often asked participants to provide inflections for nonce-words (e.g., “wug,” Berko, 1958) to eliminate the effects of lexical factors (Bybee and Moder, 1983; Prasada and Pinker, 1993; Albright and Hayes, 2003).

The goal of the present study was to explore whether agreement processes in adults could be studied in a similar way, by using items that differed systematically in grammatical structure and only minimally in lexical content. We used Staub’s forced-choice completion task and a picture description task described below. Both tasks featured a small set of high frequency words (four nouns and four color adjectives) combined into sentences such as *the circle next to star is green*, *the triangle next to the circle is red*, and so on. An obvious prediction is that the attraction effect and the attraction asymmetry seen in earlier studies should be replicated. Alternatively, one might expect that when the variability of the semantic content of the phrases is dramatically reduced, participants may focus entirely on the grammatical encoding processes and errors might therefore be rare and independent of the number specifications of the nouns.

There are two main reasons for our interest in exploring the usefulness of the paradigms described here. First, in spite of the substantial body of work on agreement, there are still many unresolved issues (for recent reviews see Bock and Middleton, 2011; Gillespie and Pearlmutter, 2011), some of which might fruitfully be addressed using lexically simple and uniform materials. Though the generation of subject-verb agreement is a grammatical process based on the number assigned to the subject noun phrase, speakers’ decisions are affected by morpho-phonological, semantic, and pragmatic variables as well (e.g., Barker et al., 2001; Hartsuiker et al., 2003; Haskell and MacDonald, 2003; Thornton and MacDonald, 2003; Solomon and Pearlmutter, 2004; Brehm and Bock, 2013; Veenstra et al., 2014a). When such variables are not of interest, it might be advisable to minimize their influence on people’s behavior by using simple and uniform materials. For instance, a much debated issue is whether and how the syntactic structure of the subject noun phrase influences the agreement process (e.g., Bock and Cutting, 1992; Franck et al., 2002; Badecker and Kuminiak, 2007; Gillespie and Pearlmutter, 2013). The existing evidence on this issue is inconsistent and, in our view, difficult to evaluate because the relevant studies have used different materials and, at times, different languages. Thus, it is possible that semantic or pragmatic variables concealed or augmented effects of syntactic structure in some of the relevant studies. Effects of syntactic structure on agreement processes might surface more clearly when other influences on the agreement process are minimized.

To give another example, Solomon and Pearlmutter (2004) have proposed that agreement processes are affected by the time course of noun phrase planning, with parallel planning of the two nouns leading to more interference of their number features and hence an increased likelihood of errors. Assessing this hypothesis requires paradigms where the time course of the retrieval of the two nouns is tightly controlled such that the retrieval processes either do or do not overlap. We have demonstrated recently that control over the time course of retrieval can be achieved by using a small set of items with similar retrieval times for all head and local nouns in a condition (Veenstra et al., 2014b).

A second reason to favor the development of agreement paradigms using lexically simple material comes from the desire to gain insight about grammatical encoding processes by expanding the study of agreement to different populations. Current studies on agreement (and language production generally) are conducted almost exclusively on highly educated young adults, in only a minute subset of the world’s languages. To the best of our knowledge, there are no systematic studies of the development of agreement processes in children, or of effects of literacy or mere print exposure on agreement processes. Furthermore, there are but a handful of studies that extend the study of agreement beyond English, Dutch, French, or Italian [Badecker and Kuminiak, 2007 (Slovak); Lorimor et al., 2008 (Russian); Deutsch and Dank, 2009 (Hebrew); Mirkovic and MacDonald, 2013 (Serbian)]. For research in these areas, and in particular for comparisons of agreement processes across groups and/or languages, it would be useful to develop sets of materials consisting of frequent words. Such materials are suitable for studies involving participants with little or no reading and restricted vocabularies, and could be readily translated between languages for cross-linguistic comparison. Finally, to go beyond descriptive work and to link differences between groups or individuals in agreement skills to educational or cognitive variables (such as executive control or working memory), agreement skills need to be assessed in an efficient and reliable way. High reliability may be easier to achieve when the items are similar in lexical content than when they are more variable.

In short, using simple and uniform materials may be advisable whenever researchers want to focus study on the grammatical components of the agreement processes. Against this, one may argue that the tools to be developed here, reliable as they may be, are unlikely to have any validity for assessing grammatical processing in natural speech. Although we find it unlikely that the processes underlying agreement should be fundamentally different in lexically reduced vs. more enriched contexts, this is an empirical issue for which the current paradigm could be modified (see General Discussion). More importantly, however, one could say that grammatical encoding processes cannot be separated from conceptual and lexical retrieval processes, and therefore the development of methods to isolate agreement processes is pointless. We are sympathetic to views that stress that conceptual, lexical, and grammatical processes are tightly linked in both speech comprehension and production (for recent discussion, see Elman, 2009; Borovsky et al., 2012; Fedorenko et al., 2012; Gennari et al., 2012; Konopka and Meyer, 2014). Nevertheless, it seems likely to us that one consequence of learning a language is to abstract away from the contexts in which utterances occur, that is, to learn the “rules” of a language. Although context is demonstrably important for how people produce and comprehend language, speakers nonetheless know the grammatical rules of their language, including those pertaining to agreement, and can apply them to express novel ideas in novel combinations of words. In this sense, agreement skills are real and distinguishable from the knowledge of individual words and the message-level contexts in which they occur. Whether the application of this knowledge is probabilistic or deterministic is beyond the scope of the current work.

Beyond issues of the multiple constraints that influence the agreement process is the need to access the processes of agreement while minimizing the need to use comprehension to first generate a to-be-produced message. Almost all of the agreement studies described above have used variants of the sentence completion paradigm. An attractive feature of this paradigm is that the characteristics of the subject phrase can be perfectly controlled. However, the task is not a pure production task, and includes comprehension and working memory components as well. For many purposes, this is unproblematic, especially since there is strong evidence that the grammatical encoding processes in both tasks are likely to be similar (Pearlmutter et al., 1999; Tooley and Bock, 2013). However, the time course of creating the grammatical and conceptual structure underlying subject noun phrases is likely to be different when participants read noun phrases relative to when they generate them themselves on the basis of conceptual information. These differences may, in turn, affect the processes involved in generating subject verb agreement. If the research goal is to investigate the processes of grammatical encoding in production, it may sometimes be desirable to minimize the comprehension component. This goal can, at least for some types of materials, be achieved by using picture description tasks.

Picture description has recently been used to study agreement in experiments by Gillespie and Pearlmutter (2011), who investigated the effect of semantic integration on attraction (for other studies about semantic integration, see Solomon and Pearlmutter, 2004; Brehm and Bock, 2013; Veenstra et al., 2014a). Participants saw displays with two pictures, one of which was to be named as the head noun and the other the local noun of a subject phrase. One picture had a colored outline, indicating that it was to be used as the head noun. The color of this outline determined which preposition participants had to use to link the two nouns. Blue indicated *for*, yielding integrated phrases such as *the apple for the pie(s)*; green indicated *near*, yielding unintegrated phrases such as *the apple near the pie(s)*. These subject phrases were then completed to full sentences. Results of this study showed the grammatical attraction effect, but no effect of the prepositions.

In Experiment 1 of the present study, we used a simpler picture description task: upon seeing a configuration of colored geometrical figures, participants produced sentences such as *the star next to the circles is blue*. The number of objects was varied across items in order to elicit subject noun phrases with singular and plural head and local nouns. We investigated whether these simple materials would induce a grammatical attraction effect, such that there would be more subject-verb agreement errors when the two nouns mismatched than when they matched in number. It is not self-evident that a replication of this key finding from the literature would be obtained in this task. Given that the visual and conceptual processes of the displays and the retrieval of the object names were very simple, adult participants might make very few agreement errors.

As shown in **Table 1**, we used two sets of displays: one with overlapping pictures, to be described using *met* (*with*), and one with non-overlapping pictures, to be described using *naast* (*next to*). This allowed us to examine whether the spatial arrangement of the pictures (or the preposition used to link the head and local noun) affected attraction. Earlier studies have shown that the semantic relationship between the head and local noun varied, for instance, in pairs such as *the driver with/for the actor(s)* or *the bowl with the stripe(s)/spoon(s)*, and can influence the generation of agreement (see Brehm and Bock, 2013; Veenstra et al., 2014a). Such studies have shown that after subject phrases where the head and local noun are conceptually tightly linked (e.g., *the driver for the actor*, *the bowl with the stripe*), fewer agreement errors are made relative to subject phrases with weakly linked head and local nouns (e.g., *the driver with the actor*, *the bowl with the spoons*; but see Solomon and Pearlmutter, 2004, for a different pattern of results). In addition, Humphreys and Bock (2005) found effects of implied spatial relations on agreement, with more plural verbs chosen for spatially separated phrases (e.g., *the gang on the motorcycles*) than for the spatially collected phrases (e.g., *the gang near the motorcycles*). We explored whether differences in the spatial arrangements of the objects had similar effects. If so, the attraction effect should be stronger for the items featuring spatial separation of the objects (the *naast*-items) than for the items featuring spatially integrated objects (the *met*-items).

**Table 1:**
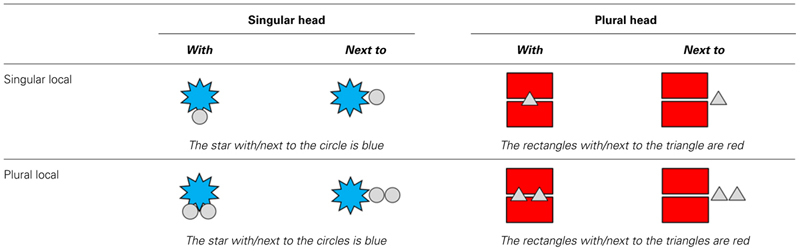
An example of pictures in eight conditions in Experiment 1.

Experiment 1 used a picture description task. In Experiment 2, we used Staub’s forced-choice completion task (Staub, 2009, 2010; Veenstra et al., 2014a) with corresponding materials to determine whether the results seen in the picture description task would be replicated. If the current paradigm captures critical aspects of the agreement process, we predict that agreement errors should be more likely when nouns mismatch relative to when they match, and that this pattern should be larger for sentences beginning with singular head nouns. Furthermore, the reaction times (RTs, Experiment 2) should show parallel patterns, with slower RTs for mismatching conditions, and a larger mismatch effect for sentences beginning with singular head nouns.

## EXPERIMENT 1

### METHODS

#### Participants

Twenty-nine native speakers of Dutch, most of them university students, participated after giving written informed consent. Approval to conduct this study was given by the Ethics Board of the Social Sciences Faculty of Radboud University, Nijmegen. Data from one participant were excluded because they did not use verbs in their descriptions. Of the remaining 28 participants, 22 were female (mean age = 20.7 years). All participants in this study only took part in one of the experiments.

#### Design and materials

The experiment had a 2 (Head Noun Number: singular/plural) by 2 (Local Noun Number: singular/plural) by 2 (Preposition: *with /next to*) factorial design. Each subject phrase consisted of a determiner and a head noun (singular or plural) followed by a preposition (*met/with* or *naast/next to*), which was then followed by a determiner and a local noun (singular or plural). Only common nouns were used, which take the number-ambiguous determiner *de*. Specifically, we used four simple shapes (*cirkel*, *driehoek*, *ster*, *rechthoek*; English: *circle*, *triangle*, *star*, *rectangle*). This led to subject phrases such as *de ster naast de cirkels/the star next to the circles* (see **Table 1**).

Pictures varied in size from 224 × 224 pixels to 256 × 509 pixels, corresponding to 6° to 13° of visual angle. Four colors were used (blue, red, yellow, and green), resulting in a total of 64 items in eight conditions. The resulting 512 trials were divided over four lists. In every list, each noun appeared 64 times as a head noun and 64 times a local noun. Each color appeared 64 times, and each preposition 128 times. The experiment consisted of four experimental blocks and two practice blocks consisting of 40 random experimental displays^1^.

#### Procedure

Participants were tested individually in a soundproof booth. The participants were instructed to give descriptions of the pictures with the following construction: *the* (*colored shape, head noun*) *with/next to the* (*gray shape, local noun*) *is/are* (*color*). They were instructed to use *with* when the shapes on the screen overlapped and to use *next to* when they were positioned next to each other. This is fully consistent with the use of the two prepositions in everyday language. Participants were told that their focus throughout the experiment should be on the correct names for the shapes. Then they were familiarized with the task and the pictures in two practice blocks of 20 trials each, which took about 3 min to administer.

On each trial a fixation cross was presented 200 pixels left from the center of the screen at 0.4° visual angle for 500 ms, followed by a blank screen of 150 ms. Then the picture was presented in the center of the screen for 2750 ms. Descriptions had to be given within a time limit of 2750 ms, which was indicated at the top of the screen with a timer. After 2750 ms, the picture disappeared and a blank screen appeared for another 500 ms. Responses were recorded for 3900 ms from the onset of the picture.

#### Scoring and analysis

The participants’ responses were scored online by the experimenter and later checked oﬄine. Responses were coded as correct, as featuring subject-verb agreement errors, or miscellaneous errors (incorrect or missing object names or numbers, colors or prepositions).

Following recent studies on agreement, statistical analyses were conducted using linear mixed effects regression models (e.g., Staub, 2009; Brehm and Bock, 2013; Gillespie and Pearlmutter, 2013; Veenstra et al., 2014a). The analyses were run in R version 2.14 using linear mixed effects models with crossed effects of subjects and items using the lme4 package (Bates, 2005; R Development Core Team, 2011). In order to avoid collinearity and to maximize the likelihood of model convergence, the variables Mismatch, Block, Preposition, and Head Noun Number were mean centered prior to analysis (Baayen, 2008). Given the coding used, negative regression coefficients correspond to more errors for number match, earlier blocks, the preposition *with*, and singular head nouns.

The fixed effects in the models included Head Noun Number (singular vs. plural), Mismatch (between the head and local noun number: yes vs. no), Preposition (*with* vs. *next to*), and Block (1 through 4). The list participants saw was initially included as a fixed effect, but as it did not contribute significantly to any of the models, we collapsed across this factor. Random intercepts were included for subjects and items, as well as random slopes to subjects and items for Head Noun Number, Mismatch, Preposition, and Block. Model selection started with a full model, leaving out non-significant interactions with each step, after which the model was tested for complexity (as measured with AIC/BIC). Maximal random slopes were included where possible (Barr et al., 2013). Main factors were kept for theoretical reasons. Error rates were analyzed using a logistic linking function (Jaeger, 2008).

Participants’ response times were not analyzed, as the critical part of the sentence (the verb) did not appear sentence-initially and the difficulty of the agreement processes was unlikely to be reflected in the sentence onset latencies.

### RESULTS

**FIGURE 1:**
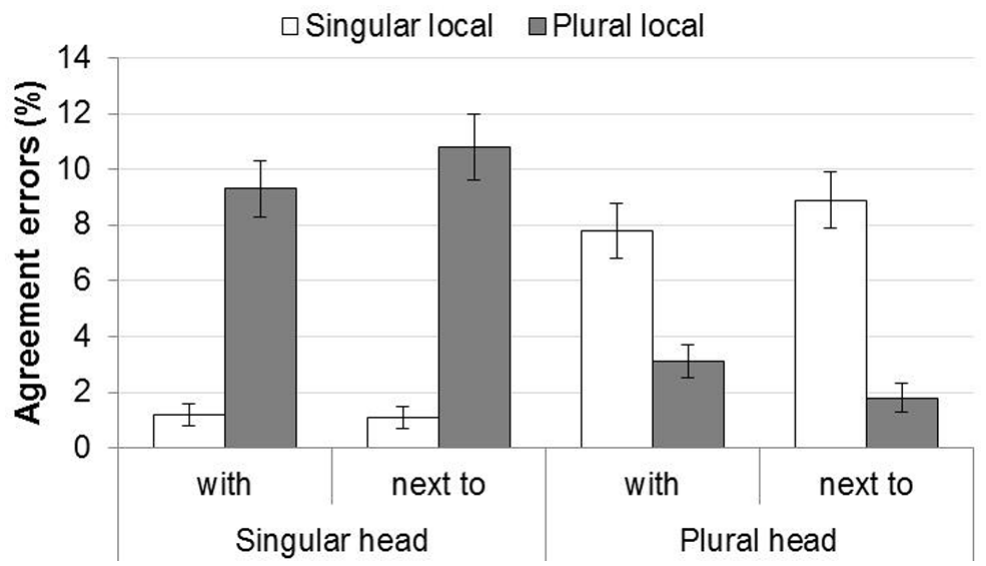
**Agreement errors in Experiment 1.** Error bars show the SE of the mean across participants, for illustrative purposes.

Miscellaneous errors occurred on 15.8% of the trials (see **Table 2** for their distribution across conditions). **Figure 1** shows the percentage of agreement errors among the remaining responses.

**Table 2:** Percentage of miscellaneous errors per condition.

		Preposition	
		With	Next to
Singular head	Singular local	13.7%	16%
	Plural local	13.3%	19.8%

Plural head	Singular local	14.6%	18.1%
	Plural local	13.4%	17.3%

There were clear attraction effects for both singular and plural heads. This pattern was confirmed by the statistical analysis. The regression model (see **Table 3**) showed main effects of Head Noun Number, Mismatch, and Block, but no main effect of Preposition. The main effect of Head Noun Number indicates that more errors were made for plural heads (*M* = 5.4%, SD = 22.9%) than for singular heads (*M* = 5.5%, SD = 22.5%)^2^, whereas the main effect of Mismatch indicated that more errors were made when the head and local noun number mismatched (*M* = 9.2%, SD = 28.9%) than when they matched (*M* = 1.8%, SD = 13.2%). Over the course of the experiment, participants made fewer errors, indicated by the main effect of Block. Importantly, there was an interaction between Head Noun Number and Mismatch, and follow-up analyses showed that attraction was stronger for singular heads (*M*_d_ = 8.9%, SD_d_ = 0.82%) than for plural heads (*M*_d_ = 5.9%, SD_d_ = 0.82%): singular heads combined with mismatching local nouns yield more agreement errors than those combined with matching local nouns (ß = 2.51, SE = 0.38, *p* < 0.001). This effect was weaker, but still reliable for plural heads (ß = 0.77, SE = 0.15, *p* < 0.001).

**Table 3:** Logistic mixed-effects model predicting agreement errors in Experiment 1.

Variable	Coefficient	SE	*z*-value	Pr(>|*z*|)	Random slope
(Intercept)	-4.08	0.20	-20.19	<0.001	Subjects, items
Head noun number	0.38	0.13	2.83	0.005	Subjects, items
Mismatch	1.28	0.15	8.45	<0.001	Subjects, items
Block	-0.20	0.05	-3.75	<0.001	Subjects, items
Preposition	-0.03	0.07	0.38	0.706	
Head number × mismatch	-0.52	0.13	-4.16	<0.001	

*Coefficients correspond to logits.*

### DISCUSSION

The speeded picture description task of Experiment 1 yielded three main results: first, there was a clear attraction effect: more agreement errors were made for subject phrases with mismatching head and local nouns, compared to subject phrases with matching head and local nouns. Second, the experiment replicated the attraction asymmetry seen in previous research: the attraction effect was weaker for plural heads combined with singular local nouns than for singular heads combined with plural local nouns. Unlike previous experiments using the sentence completion paradigm, however, the attraction effect observed for plural head nouns combined with singular local nouns was reliable. Third, there was no effect of preposition, as equal proportions of agreement errors were made for sentences with *met* (*with*) and with *naast* (*next to*). One might have expected that the difference in spatial arrays (with overlapping vs. separate objects) and the associated use of prepositions could affect the generation of agreement, similar to the effect of semantic integration. This expectation was not borne out.

## EXPERIMENT 2

The second experiment used the forced-choice task developed by Staub (2009, 2010; see also Veenstra et al., 2014a). The written subject phrases corresponded to the intended descriptions of the pictures in Experiment 1. The forced-choice task has the advantage that response times for verb selection can be measured. We predicted a replication of the results from Experiment 1, with an attraction effect and an asymmetry in the attraction effect in the error rates and parallel patterns in the response times.

### METHODS

#### Participants

Thirty-one native speakers of Dutch participated after giving written informed consent. Data from three participants were excluded due to poor performance on the catch trials (see below). Of the remaining 28 participants, 22 were female (mean age = 22.4 years).

#### Design and materials

The materials were identical to Experiment 1, but instead of pictures, participants saw written subject phrases, see **Table 4**. Whereas Staub (2009, 2010) presented his participants with *is/are*, the participants of the present study saw full verb phrases, such as *is blue/are blue*. This was done in order to match the sentences to those of Experiment 1, where speakers produced full sentences.

**Table 4:** An example item in eight conditions.

		Preposition	
		With	Next to
Singular head	Singular local	*The star with the circle*	*The star next to the circle*
	Plural local	*The star with the circles*	*The star next to the circles*

Plural head	Singular local	*The stars with the circle*	*The stars next to the circle*
	Plural local	*The stars with the circles*	*The stars next to the circles*

Sixty-four filler items were constructed with different structures, such as *the star or the circle*, or *the star and the circle*, to prevent participants from basing their answer solely on the number of the first noun.

One potential strategy in which participants might engage is to only pay attention to the head noun as selection of the correct verb phrase depends on this noun. In order to prevent such a strategy from occurring, and to encourage participants to carefully process the entire subject noun phrases, catch trials were included that required participants to repeat the noun phrases and complete them with a spoken continuation (see Procedure). This same procedure has been used successfully before in earlier studies (Brehm and Bock, 2013; Veenstra et al., 2014a). As participants could not predict which trials would be catch trials, they had to pay close attention to the wording of all subject phrases.

A practice block of 10 trials (consisting of random experimental trials) was added to each list. Items were presented in a fixed random order. As in Experiment 1, the practice items were repeated in the experimental blocks.

#### Procedure

Participants were tested individually in a sound-proof booth in front of a computer. First, a fixation cross appeared in the center of the screen for 1000 ms at 0.4° visual angle. Then the subject phrase was presented in the center of the screen in a word-by-word fashion. Each word appeared for 250 ms, followed by a blank screen for 150 ms. After presentation of the subject phrase, a screen with two verb phrases appeared; the singular option (e.g., *is blauw*) on the left and the plural option (e.g., *zijn blauw*) on the right. Participants were instructed to press the corresponding button on a two-button button box as quickly as possible. Feedback was provided to incorrect answers [the word *fout* (*wrong*) appeared for 1500 ms]. When the answer was correct, the next trial followed after a blank screen shown for 1500 ms.

Catch trials had a structure similar to that of experimental trials, except that instead of the screen with two verb phrase options, the word *herhaal* (*repeat*) appeared, prompting participants to repeat the subject phrase and complete the sentence aloud freely. Answers were recorded for 3000 ms. The experiment consisted of a practice block of 10 trials and 4 experimental blocks of 64 experimental, 8 catch and 16 filler trials each.

#### Scoring and analysis

Catch trials were analyzed only in order to check participants’ attention to the subject phrases. Three participants made over 15% errors on catch trials, usually failing to repeat the subject phrases correctly. Their data were excluded from further analysis as the high number of repetition errors raised doubts about their processing of the subject phrases on experimental trials. The responses on the experimental trials were coded for accuracy and response time. Analyses below concern the experimental trials only.

Trials in which an answer was given faster than 200 ms were excluded from the analysis (3.9% of the data). On these trials, participants may have decided on their answer before the sentence was completed, possibly limiting the influence of the local noun.

Only correct responses on experimental trials were included in the analysis of response times. A histogram showed that the distribution of response times was rightward skewed; therefore, the analyses were performed on natural log-transformed response times. Response times more than three standard deviations above the participant’s mean were excluded (1.5% of the data). The inclusion of random slopes in the analysis of response times meant that resampling methods for calculating statistical probability were not available. Thus, factors were judged significant when the absolute *t*-value exceeded 2 (Baayen, 2008).

The statistical analyses of agreement errors were identical to Experiment 1.

### RESULTS

#### Agreement errors

**FIGURE 2:**
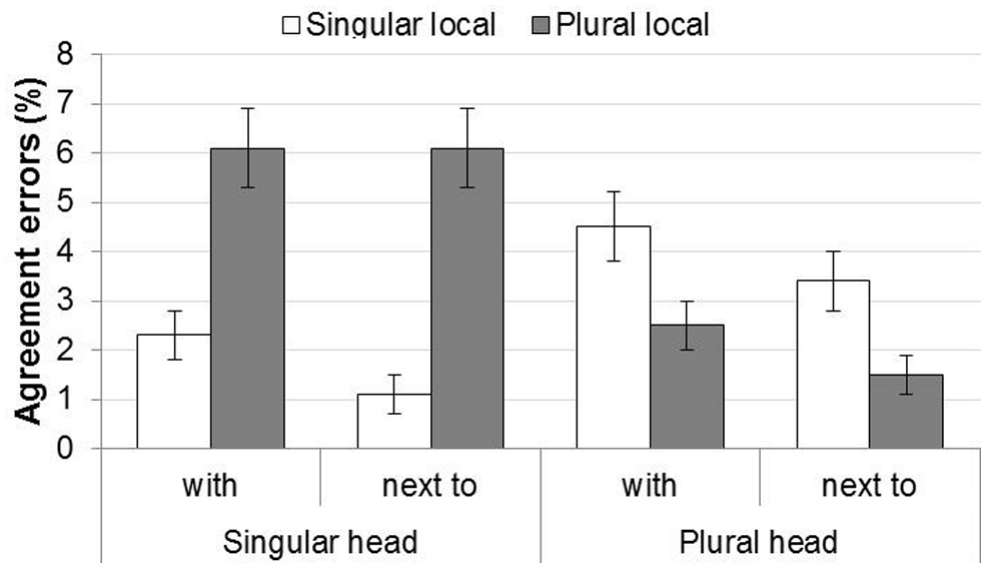
**Agreement errors in Experiment 2.** Error bars show the SE of the mean across participants, for illustrative purposes.

Agreement errors consisted of plural answers given to trials with a singular head noun and singular answers given to trials with a plural head noun. The proportions of agreement errors are shown in **Figure 2**.

The figure shows that there was attraction for both singular and plural head nouns, and this effect was stronger for singular head nouns than for plural head nouns (i.e., the attraction asymmetry). The preposition *met* led to more errors than *naast.* These patterns were confirmed by the statistical analysis, see **Table 5**.

**Table 5:** Logistic mixed-effects model predicting agreement errors in Experiment 2.

Variable	Coefficient	SE	*z*-value	Pr(>|*z*|)	Random slope
(Intercept)	-4.15	0.19	-22.17	<0.001	Subjects, items
Head noun number	<0.001	0.10	0.02	0.984	Subjects, items
Mismatch	0.38	0.11	3.50	<0.001	Subjects, items
Preposition	-0.20	0.09	-2.37	0.017	Subjects, items
Block	-0.39	0.07	-5.42	<0.001	Subjects, items
Head number × mismatch	-0.24	0.09	-2.65	0.007	

*Coefficients correspond to logits.*

The statistical analysis showed main effects of Mismatch, Preposition, and Block. The main effect of Mismatch shows that items with mismatching head and local nouns yielded more errors (*M* = 5%, SD = 21.8%) than items with matching head and local nouns (*M* = 1.9%, SD = 13.5%). The main effect of Preposition arose because there were more errors for *met*-items (*M* = 3.9%, SD = 19.3%) than *naast*-items (*M* = 3.0%, SD = 17.1%). The effect of Block was due to the fact that participants made fewer errors over the course of the experiment. Importantly, the analysis also showed a Mismatch by Head Noun Number interaction. This result was followed up with separate analyses for singular and plural heads. The mismatch effect was significant for singular heads (*M*_d_ = 4.4%, SD_d_ = 0.64; ß = 0.64, SE = 0.16, *p* < 0.001), but unlike the results seen in Experiment 1, was not significant for plural heads (*M*_d_ = 1.9%, SD_d_ = 0.57; ß = 0.14, SE = 0.14, *p* = 0.327). This pattern thus replicates the classic attraction asymmetry observed in previous studies using the sentence completion paradigm.

#### Response times

**FIGURE 3:**
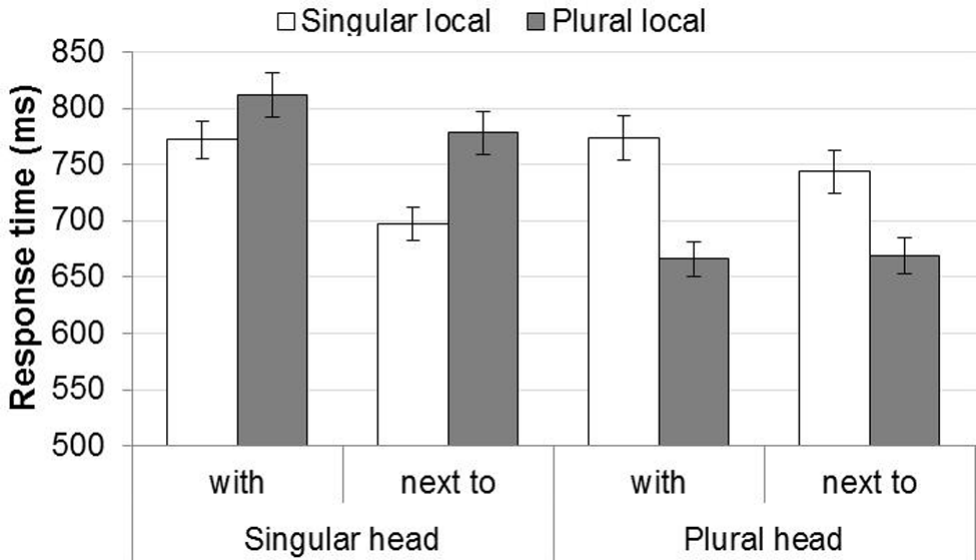
**Response times in Experiment 2.** Error bars show the SE of the mean across participants, for illustrative purposes.

The response times showed roughly the same pattern as the agreement errors, see **Figure 3**.

The statistical analysis revealed no significant interactions, only main effects of Head Noun Number, Mismatch, Preposition, and Block (see **Table 6**). The main effect of Head Noun Number came from slower responses in choosing the verb phrase when the head noun was singular (*M* = 764 ms, SD = 510 ms) than when it was plural (*M* = 713 ms, SD = 501 ms). The main effect of Mismatch shows that participants were slower when the numbers of the head and local noun mismatched (*M* = 777 ms, SD = 551 ms) compared to when they matched (*M* = 701 ms, SD = 455 ms). The effect of Preposition came from slower response times when the item contained *met* (*M* = 755 ms, SD = 517 ms) relative to when it contained *naast* (*M* = 721 ms, SD = 494 ms). Finally, participants became faster over the course of the experiment, as indicated by the effect of Block. In contrast to the error rates, there was no interaction between Head Noun Number and Mismatch, thus no evidence of an attraction asymmetry.

**Table 6:** Logistic mixed-effects model predicting response times in Experiment 2.

Variable	Coefficient	SE	*t*	Random slope
(Intercept)	6.41	0.08	81.65	Subjects, items
Head noun number	-0.03	0.01	-3.43	Subjects, items
Mismatch	0.04	0.01	4.08	Subjects, items
Preposition	-0.02	0.01	-2.14	Subjects, items
Block	-0.09	0.01	-7.97	Subjects, items

### DISCUSSION

The forced-choice sentence completion task of Experiment 2 yielded three main results. First, there was a clear attraction effect, with more agreement errors for subject phrases with mismatching head and local nouns than for subject phrases with matching head and local nouns. In addition, there was an attraction effect in the response times: participants took longer to choose a verb when the number of the nouns mismatched, than when it matched.

Second, the error rates showed the classic attraction asymmetry as the attraction effect was significant for singular heads combined with plural local nouns, but not for plural heads combined with singular local nouns. In contrast, response times showed no such asymmetry: singular and plural head nouns yielded reliable attraction effects of similar magnitude.

Third, there was a main effect of preposition for error rates and response times. Higher error rates and slower responses for the *met*-items than for the *naast*-items suggested that the phrases featuring *met* were more difficult. Given that no difference between the prepositions was seen in Experiment 1, this effect may be due to the fact that the meaning of *naast* is more well-defined than that of *met.* The same holds for English *next to* and *with*: a phrase such as *the star next to the circle* clearly indicates spatial separation, whereas *the star with the circle* might be interpreted to mean that the star is adorned with a circle or that it is next to the circle. This ambiguity may have created some confusion and interfered with the selection of the correct verb form. In Experiment 1, where the participants saw displays of the target objects, no such ambiguity arose and therefore there was no effect of preposition on the error rates.

Note that the main effects of preposition seen in the current experiment do not match the effects of semantic integration or spatial distribution observed in previous studies (Solomon and Pearlmutter, 2004; Humphreys and Bock, 2005; Brehm and Bock, 2013; Veenstra et al., 2014a). Based on the earlier results one would expect more agreement errors or a stronger attraction effect for singular head nouns in *next to*-items compared to the *with*-items. This is because *next to* highlights the presence of several distinct objects, whereas a noun phrase featuring *with* can be interpreted as referring to a single object (e.g., a circle adorned with a star). In contrast to these predictions, we found that the participants made fewer agreement errors on *next to* than *with* items, presumably because of the ambiguity of *with*.

## GENERAL DISCUSSION

The current study examined the production of subject-verb agreement in two paradigms: a picture description task in Experiment 1 and a forced-choice sentence completion task in Experiment 2. The experiments differed from previous experiments of agreement in the choice of materials, which were kept very simple. In the picture description task, participants saw different combinations of four geometrical figures shown in four colors and described them in sentences such as *the star next to the circles is blue*. In the forced-choice sentence completion task, they read noun phrases featuring the same object names and chose the correct verb forms and color adjectives. Our main goal was to explore whether the generation of agreement in adults could be investigated using such simple materials. To this end, we examined whether attraction and the attraction asymmetry, key findings reported in all published studies of agreement, would be replicated with our materials. Results across both studies showed that we were able to replicate critical patterns of attraction using these simple materials. We first discuss the theoretical implications of the present results and then turn to methodological issues.

Attraction is the observation that agreement errors are more likely when the head noun and the following local noun in a subject noun phrase mismatch in number relative to when they match (Bock and Miller, 1991; Bock and Eberhard, 1993; Vigliocco et al., 1995; Bock et al., 1999; Haskell and MacDonald, 2005). Our results are clear-cut: in both experiments, reliably more agreement errors occurred for mismatching than for matching head and local nouns. Additionally, response times for correct trials in Experiment 2 were longer when the head and local noun mismatched than when they matched, indicating increased difficulty to compute agreement in the presence of an interfering local noun. In sum, both experiments yielded evidence for attraction. This finding represents initial evidence that agreement processes in adults can be studied with simple and repetitive materials.

As noted above, earlier studies have also found an asymmetry in the attraction effect, with the effect far stronger for singular than plural head nouns (Bock and Miller, 1991; Bock and Eberhard, 1993; Vigliocco et al., 1995; Bock et al., 1999; Haskell and MacDonald, 2005; but see Franck et al., 2002, 2006). In both of our experiments, the error rates showed such an asymmetry, though in Experiment 1 the attraction effect was significant for both singular and plural heads. The response latencies in Experiment 2 did not show an attraction asymmetry. Overall, then, our data show a weaker attraction asymmetry than one might have expected based on previous research. In earlier work, the attraction asymmetry has often been accounted for by reference to the concept of markedness (e.g., Eberhard, 1997; Eberhard et al., 2005): singular nouns are unmarked, whereas plural nouns are marked, thus, only features from the latter can interfere with computing the inflection of the verb. Given that we found an attraction effect with singular local nouns, our data suggest that the effect of markedness on the generation of agreement may be graded rather than categorical, with marked plural local nouns exerting a stronger effect on the choice of the verb form than unmarked singular local nouns (for similar conclusions, see Haskell et al., 2010; Hanke et al., 2013). The attraction asymmetry thus continues to serve as an important testing ground for theories about the processes and representations underlying agreement. The fact that agreement errors from singular local nouns can reliably elicit attraction in the picture naming paradigm developed here suggests that this paradigm should prove useful to address issues of markedness in future investigations.

The main goal of the present study, however, was a methodological one, namely to explore how well agreement processes could be studied when the lexical content of the utterances was reduced to a minimum. We did this in two paradigms, the forced-choice completion paradigm and the picture description paradigm. Turning first to the comparison of the two paradigms, it is evident that each of them has advantages and disadvantages, and that consequently, their relative usefulness will depend on the research question and experimental context. Advantages of the forced-choice paradigm are that the materials are easy to generate, and that the responses are fast to code. Furthermore, data loss due to invalid responses is minimal, and perhaps most importantly, response times for the choice of the verb form can readily be obtained. A potential disadvantage is that the task is not a pure production task. It includes a comprehension component as the participants have to read or listen to the preambles. The picture description task, in contrast, does not involve such a comprehension component, and the task gets closer to requiring participants to generate their own message. However, the materials for a picture description experiment are slightly more difficult to generate, there is likely to be more data loss due to invalid responses, and coding the responses and measuring response latencies is more time-consuming. Data loss in a picture description task with simple materials can, however, be substantially lower than reported for some classic free preamble completion tasks (e.g., 20% in this study compared to 40–75% in Bock and Miller’s, 1991 study).

Turning to the materials, the practical advantages of using small sets of items that are repeated many times over the course of the experiment might also be obvious. Small item sets featuring simple pictures and high frequency words are easy to generate. In a picture description task, there will be little data loss due to invalid nouns being produced since the descriptive task is easy and repeated many times across trials. Furthermore, the coding of the responses is likewise relatively straightforward.

More importantly, there is little room for conceptual and lexical variables to affect the participants’ responses. As mentioned in the Introduction, most studies of agreement have used parallel versions of the subject noun phrases [e.g., *the bridge to the island(s)*] in different conditions so that the conditions were well matched for lexical content. Significant variability in semantic content across items is usually allowed. By contrast, the items in the simple materials used here are extremely similar. The variance in the participants’ response speed and accuracy due to differences between the items in semantic content or due to interactions of item-specific semantic effects with other variables must be lower than in studies using larger and more heterogeneous sets of items. This reduction in variance should facilitate detecting effects of the manipulation of grammatical structure.

As already discussed in the Section “Introduction,” picture description and sentence completion experiments can be viewed as tests of the participants’ agreement skills. One would expect the reliability of an agreement test to increase as variability in the semantic content of the items decreases. To assess whether this was the case, we computed the split-half reliability (the first 64 trials vs. the second 64 trials) for the mismatch effect in the response latencies in Experiment 2 of the current study and for a similar experiment using different lexical items on each trial (Experiment 2, Veenstra et al., 2014a). As that study only employed singular head nouns with matching and mismatching local nouns, we only included the trials with singular heads from the current study in the reliability analysis. The two experiments were similar in the number of items and participants. For Experiment 2 of the present study, the correlation in the effect sizes was *r* = 0.74 (Cronbach’s α = 0.82); thus, participants who had small or large mismatch effects in the first half of the experiment tended to have small or large effects in the second half as well. By contrast, in our earlier study, the corresponding correlation was only *r* = 0.16 (Cronbach’s α = 0.27). Interestingly, the split-half reliability for the mismatch effect in the error rates was high in both experiments: *r* = 0.71 (Cronbach’s α = 0.75) in the present study and *r* = 0.80 (Cronbach’s α = 0.89) in Veenstra et al. (2014a); the higher reliability is likely due to the relatively low error rates in the latter study. Nevertheless, the point remains that the lexical content of the items can have a substantial impact on the participants’ responses. In order to assess grammatical encoding skills in an individual or a group of participants, one might therefore want to minimize lexical variability.

Of course, the most important criterion in evaluating an experimental paradigm is whether it can be used to address practically or theoretically important issues. Whether this is the case for the methods described here needs to be determined in future research. We think that in studying grammatical encoding the use of lexically simple materials may prove to be beneficial. This should hold not only for research into agreement but also, for instance, for research into the generation of different syntactic structures, such as questions, relative clauses, or passive forms. Whenever the goal is to assess grammatical encoding skills in an individual (e.g., a patient) and whenever groups (e.g., young and older persons, L1 and L2 speakers of a language) are to be compared with respect to these skills, it would seem useful to use methods that measure these skills as purely and reliably as possible. The same holds for cognitive neuroscience studies aiming to understand the brain networks involved in grammatical encoding (see Segaert et al., 2012, for a study using relatively simple material to investigate syntactic priming).

One advantage of the basic paradigms used here is that they can be modified in many ways to allow researchers to address different questions or test different groups of participants. For instance, both the picture description and the forced-choice completion paradigm can be readily adapted for use in cross-linguistic research. Furthermore, as attraction was found with small item sets, the tasks may be well suited for use in persons with limited vocabularies. For instance, the materials can be adapted to include specific words that exist in the vocabulary of young children or a specific aphasic patient. In addition, the picture description task may be useful to assess agreement in groups with low literacy or persons with reading difficulties, and in persons with verbal working memory or comprehension deficits, who might struggle to understand and retain spoken preambles.

In evaluating the potential of simple materials to assess specific theoretical issues, such as the impact of the hierarchical and linear distance between the head and local noun on agreement processes, one should also keep in mind that lexically simple materials can still be grammatically complex (as in *the triangles that the dot above the circle touched are blue*). Moreover, the current paradigm would afford a gradual building-up of research into how conceptual and lexical variables influence grammatical encoding by systematically re-introducing these variables into the materials. One could, for instance, use a small set of items to investigate whether a semantic relationship between the head noun and the local noun affects the processing of agreement, or whether the animacy of nouns or their frequency matters. It is, of course, also possible to investigate the effects of the number of items and their repetition on grammatical encoding processes. The current paradigm thus affords multiple opportunities for systematically varying factors that may influence the agreement process, and serves as the starting point of research programs addressing many issues in grammatical encoding. A good general research strategy for any area of grammatical encoding might be to start simple – using small sets of repeated items – and to systematically increase the variability of the items.

## CONCLUSION

Experimental studies of grammatical encoding have often used large sets of stimuli varying widely in lexical content. Such variability might unnecessarily complicate the generation of experimental materials and, more importantly, the interpretation of the results. The current study demonstrates that reliable measures of grammatical encoding in production can be elicited using lexically simple materials. We encourage psycholinguists to explore the use of simple and homogeneous materials in studies of grammatical encoding. The present study illustrates how this can be done.

## Conflict of Interest Statement

The authors declare that the research was conducted in the absence of any commercial or financial relationships that could be construed as a potential conflict of interest.

## Footnotes

1 Additional analyses excluding the repeated displays yielded almost identical error rates; all differences in error rates per condition were less than 0.003. Thus, there was no effect of repeating some of the experimental displays during practice and testing.

2 Note that the means reported here are in the opposite direction of the model estimate of the effect of Head Noun Number. This difference is a result of variability across subjects and items that was accounted for in the random slopes of the mixed effects model. When random intercept terms alone are modeled, no main effect of Head Noun Number emerges.

## References

[B1] Albright A. Hayes B. (2003). Rules vs. analogy in English past tenses: a computational/experimental study. *Cognition* 90 119–161 10.1016/S0010-0277(03)00146-X 14599751

[B2] Baayen R. H. (2008). *Analyzing Linguistic Data: A Practical Introduction to Statistics*. Cambridge: Cambridge University Press. 10.1017/CBO9780511801686

[B3] Badecker W. Kuminiak F. (2007). Morphology, agreement and working memory retrieval in sentence production: evidence from gender and case in Slovak. *J. Mem. Lang.* 56 65–85 10.1016/j.jml.2006.08.004

[B4] Barker J. Nicol J. Garrett M. (2001). Semantic factors in the production of number agreement. *J. Psycholinguist. Res.* 30 91–114 10.1023/A:1005208308278 11291185

[B5] Barr D. J. Levy R. Scheepers C. Tily H. J. (2013). Random effects structure for confirmatory hypothesis testing: keep it maximal. *J. Mem. Lang.* 68 255–278 10.1016/j.jml.2012.11.001 PMC3881361 24403724

[B6] Bates D. M. (2005). Fitting linear mixed models in R: using the lme4 package. *R News* 5 27–30

[B7] Berent I. Pinker S. Tzelgov J. Bibi U. Goldfarb L. (2005). Computation of semantic number from morphological information. *J. Mem. Lang.* 53 342–358 10.1016/j.jml.2005.05.002

[B8] Berko J. (1958). The child’s learning of English morphology. *Word* 14 150–177

[B9] Bock K. (2004). Psycholinguistically speaking: some matters of meaning, marking, and morphing. *Psychol. Learn. Motiv.* 44 109–144 10.1016/S0079-7421(03)44004-8

[B10] Bock K. Cutting J. C. (1992). Regulating mental energy: performance units in language production. *J. Mem. Lang.* 31 99–127 10.1016/0749-596X(92)90007-K

[B11] Bock K. Eberhard K. M. (1993). Meaning, sound and syntax in English number agreement. *Lang. Cogn. Process.* 8 57–99 10.1080/01690969308406949

[B12] Bock K. Nicol J. Cutting J. C. (1999). The ties that bind: creating number agreement in speech. *J. Mem. Lang.* 40 330–346 10.1006/jmla.1998.2616

[B13] Bock K. Middleton E. L. (2011). Reaching agreement. *Nat. Lang. Linguist. Theory* 29 1033–1069 10.1007/s11049-011-9148-y

[B14] Bock K. Miller C. A. (1991). Broken agreement. *Cogn. Psychol.* 23 45–93 10.1016/0010-0285(91)90003-7 2001615

[B15] Borovsky A. Elman J. L. Fernald A. (2012). Knowing a lot for one’s age: vocabulary skill and not age is associated with anticipatory incremental sentence interpretation in children and adults. *J. Exp. Child Psychol.* 112 417–436 10.1016/j.jecp.2012.01.005 22632758 PMC3374638

[B16] Brehm L. Bock K. (2013). What counts in grammatical number agreement? *Cognition* 128 149–169 10.1016/j.cognition.2013.03.009 23680792

[B17] Bybee J. L. Moder C. L. (1983). Morphological classes as natural categories. *Language* 251–270 10.2307/413574

[B18] Corbett G. G. (2000). *Number.* Cambridge: Cambridge University Press. 10.1017/CBO9781139164344

[B19] Damian M. F. Vigliocco G. Levelt W. J. (2001). Effects of semantic context in the naming of pictures and words. *Cognition* 81 B77–B86 10.1016/S0010-0277(01)00135-4 11483172

[B20] Deutsch A. Dank M. (2009). Conflicting cues and competition between notional and grammatical factors in producing number and gender agreement: evidence from Hebrew. *J. Mem. Lang.* 60 112–143 10.1016/j.jml.2008.07.001

[B21] Eberhard K. M. (1997). The marked effect of number on subject–verb agreement. *J. Mem. Lang.* 36 147–164 10.1006/jmla.1996.2484

[B22] Eberhard K. M. Cutting J. C. Bock K. (2005). Making syntax of sense: number agreement in sentence production. *Psychol. Rev.* 112 531–559 10.1037/0033-295X.112.3.531 16060750

[B23] Elman J. L. (2009). On the meaning of words and dinosaur bones: lexical knowledge without a lexicon. *Cogn. Sci.* 33 547–582 10.1111/j.1551-6709.2009.01023.x 19662108 PMC2721468

[B24] Fedorenko E. Piantadosi S. Gibson E. (2012). Processing relative clauses in supportive contexts. *Cogn. Sci.* 36 471–497 10.1111/j.1551-6709.2011.01217.x 22256956

[B25] Ferreira V. S. Pashler H. (2002). Central bottleneck influences on the processing stages of word production. *J. Exp. Psychol. Learn. Mem. Cogn.* 28 1187–1199 10.1037/0278-7393.28.6.1187 12450341 PMC1864932

[B26] Franck J. Lassi G. Frauenfelder U. H. Rizzi L. (2006). Agreement and movement: a syntactic analysis of attraction. *Cognition* 101 173–216 10.1016/j.cognition.2005.10.003 16360139

[B27] Franck J. Vigliocco G. Nicol J. (2002). Subject-verb agreement errors in French and English: the role of syntactic hierarchy. *Lang. Cogn. Process.* 17 371–404 10.1080/01690960143000254

[B28] Gennari S. P. Mirkovic J. MacDonald M. C. (2012). Animacy and competition in relative clause production: a cross-linguistic investigation. *Cogn. Psychol.* 65 141–176 10.1016/j.cogpsych.2012.03.002 22537914 PMC4242715

[B29] Gillespie M. Pearlmutter N. J. (2011). “Effects of semantic integration and advance planning on grammatical encoding in sentence production,” in *Proceedings of the 33rd Annual Conference of the Cognitive Science Society* eds Carlson L. Hoelscher C. Shipley T. F. (Austin, TX: Cognitive Science Society) 1625–1630

[B30] Gillespie M. Pearlmutter N. J. (2013). Against structural constraints in subject–verb agreement production. *J. Exp. Psychol. Learn. Mem. Cogn.* 39 515 10.1037/a0029005 PMC4528622 22732035

[B31] Hanke M. Hamann C. Ruigendijk E. (2013). On the laws of attraction at cocktail parties: babble noise influences the production of number agreement. *Lang. Cogn. Process.* 28 1114–1133 10.1080/01690965.2012.696664

[B32] Hartsuiker R. J. Antón-Méndez I. van Zee M. (2001). Object attraction in subject-verb agreement construction. *J. Mem. Lang.* 45 546–572 10.1006/jmla.2000.2787

[B33] Hartsuiker R. J. Barkhuysen P. N. (2006). Language production and working memory: the case of subject-verb agreement. *Lang. Cogn. Process.* 21 181–204 10.1080/01690960400002117

[B34] Hartsuiker R. J. Schriefers H. J. Bock K. Kikstra G. M. (2003). Morphophonological influences on the construction of subject-verb agreement. *Mem. Cogn.* 31 1316–1326 10.3758/BF03195814 15058692

[B35] Haskell T. R. MacDonald M. C. (2003). Conflicting cues and competition in subject-verb agreement. *J. Mem. Lang.* 48 760–778 10.1016/S0749-596X(03)00010-X

[B36] Haskell T. R. MacDonald M. C. (2005). Constituent structure and linear order in language production: evidence from subject-verb agreement. *J. Exp. Psychol. Learn. Mem. Cogn.* 31 891–904 10.1037/0278-7393.31.5.891 16248740

[B37] Haskell T. R. Thornton R. MacDonald M. C. (2010). Experience and grammatical agreement: statistical learning shapes number agreement production. *Cognition* 114 151–164 10.1016/j.cognition.2009.08.017 19942213 PMC2812604

[B38] Humphreys K. R. Bock K. (2005). Notional number agreement in English. *Psychon. Bull. Rev.* 12 689–695 10.3758/BF03196759 16447383

[B39] Jaeger T. F. (2008). Categorical data analysis: away from ANOVAs (transformation or not) and towards logit mixed models. *J. Mem. Lang.* 59 434–446 10.1016/j.jml.2007.11.007 19884961 PMC2613284

[B40] Konopka A. E. Meyer A. S. (2014). Priming sentence planning. *Cogn. Psychol.* 73 1–40 10.1016/j.cogpsych.2014.04.001 24838190

[B41] Levelt W. J. M. Roelofs A. Meyer A. S. (1999). A theory of lexical access in speech production. *Behav. Brain Sci.* 22 1–38 10.1017/S0140525X99001776 11301520

[B42] Lorimor H. Bock K. Zalkind E. Sheyman A. Beard R. (2008). Agreement and attraction in Russian. *Lang. Cogn. Process.* 23 769–799 10.1080/01690960701774182

[B43] Mirkovic J. MacDonald M. C. (2013). When singular and plural are both grammatical: semantic and morphophonological effects in agreement. *J. Mem. Lang.* 69 277–298 10.1016/j.jml.2013.05.001 24039340 PMC3770477

[B44] Pearlmutter N. J. Garnsey S. M. Bock K. (1999). Agreement processes in sentence comprehension. *J. Mem. Lang.* 41 427–456 10.1006/jmla.1999.2653

[B45] Prasada S. Pinker S. (1993). Generalizations of regular and irregular morphology. *Lang. Cogn. Process.* 8 1–56 10.1080/01690969308406948

[B46] R Development Core Team. (2011). *R: A Language and Environment for Statistical Computing*. Vienna: R Foundation for Statistical Computing.

[B47] Schriefers H. Meyer A. S. Levelt W. J. M. (1990). Exploring the time course of lexical access in language production: picture-word interference studies. *J. Mem. Lang.* 29 86–102 10.1016/0749-596X(90)90011-N

[B48] Segaert K. Menenti L. Weber K. Petersson K. M. Hagoort P. (2012). Shared syntax in language production and language comprehension – an fMRI study. *Cereb. Cortex* 22 1662–1670 10.1093/cercor/bhr249 21934094 PMC3377967

[B49] Solomon E. S. Pearlmutter N. J. (2004). Semantic integration and syntactic planning in language production. *Cogn. Psychol.* 49 1–46 10.1016/j.cogpsych.2003.10.001 15193971

[B50] Staub A. (2009). On the interpretation of the number attraction effect: response time evidence. *J. Mem. Lang.* 60 308–327 10.1016/j.jml.2008.11.002 20126291 PMC2683024

[B51] Staub A. (2010). Response time distributional evidence for distinct varieties of number attraction. *Cognition* 114 447–454 10.1016/j.cognition.2009.11.003 20003964

[B52] Thornton R. MacDonald M. C. (2003). Plausibility and grammatical agreement. *J. Mem. Lang.* 48 740–759 10.1016/S0749-596X(03)00003-2

[B53] Tooley K. Bock J. K. (2013). On the parity of structural persistence in language production and comprehension. *Cognition* 132 101–136 10.1016/j.cognition.2014.04.002 24803423 PMC4096381

[B54] Veenstra A. Acheson D. J. Bock K. Meyer A. S. (2014a). Effects of semantic integration on subject–verb agreement: evidence from Dutch. *Lang. Cogn. Neurosci.* 29 355–380 10.1080/01690965.2013.862284

[B55] Veenstra A. Acheson D. J. Meyer A. S. (2014b). “Parallel planning and attraction in subject-verb agreement,” in *Poster Presented at the International Workshop On Language Production* Geneva

[B56] Vigliocco G. Butterworth B. Semenza C. (1995). Constructing subject-verb agreement in speech: the role of semantic and morphological factors. *J. Mem. Lang.* 34 186–215 10.1006/jmla.1995.1009

[B57] Vigliocco G. Hartsuiker R. J. Jarema G. Kolk H. H. J. (1996). One or more labels on the bottles? Notional concord in Dutch and French. *Lang. Cogn. Process.* 11 407–442 10.1080/016909696387169

